# Fishery Improvement Projects as a governance tool for fisheries sustainability: A global comparative analysis

**DOI:** 10.1371/journal.pone.0223054

**Published:** 2019-10-01

**Authors:** Beatrice Crona, Sofia Käll, Tracy Van Holt

**Affiliations:** 1 Global Economic Dynamics and the Biosphere, The Royal Swedish Academy of Sciences, Stockholm, Sweden; 2 Stockholm Resilience Centre, Stockholm University, Stockholm, Sweden; 3 NYU Stern Center for Sustainable Business, New York University, New York, New York, United States of America; Universitat Autonoma de Barcelona, SPAIN

## Abstract

Fishery Improvement Projects (FIPs) are a form of private governance using seafood supply chains to reduce environmental impacts of fishing in some of the most challenged fisheries. Some FIPs are industry-led, others are championed by NGOs. They range across many different fishery types, in both high- and low-income settings. Their diversity is notable, and their proliferation remarkable. This rapid growth suggests FIPs are becoming a key feature of the fisheries governance landscape globally. Based on a global sample of 107 FIPs, we systematically examined their reported actions, the actors involved, and their achievements in terms of policy and practice outputs. The most common actions were dialogues with policy stakeholders, data collection, and educational efforts directed at fishers. Common policy outputs included development of management plans and/or a management body, and rules for limiting entry and increasing compliance. Practice related outputs were dominated by gear changes, and observer and traceability programs. Only crab and lobster FIPs engaged in sustained policy conversations as one of the most common actions. Shrimp and tuna fisheries report more engagement in testing and implementing changes to fishery practices. While supply chain actors are involved in all FIPs, retailers and 1st tier suppliers are relatively absent from FIP activities, and are primarily involved in rallying financial support or some policy engagement. Based on our analysis we discuss the opportunities and challenges FIPs will likely need to engage with to contribute to a global transition to more socially and environmentally sustainable fisheries. We outline key areas where further work is needed to understand how FIPs can improve their contribution to global fisheries governance in the future.

## 1.Introduction

For fisheries to contribute sustainably to both environmental and social development, fisheries governance is critical [1]. However, the form such governance should take remains a key area of debate. Governance, as used here, includes the deliberation, design and implementation of rules, both formal and informal, which govern the behavior of all the actors whose actions have a bearing on fisheries outcomes [2,3]. Private governance is governance driven by various types of non-state actors, who interact to produce institutional arrangements that structure and direct actors’ behavior in a particular domain [4]. The Marine Stewardship Council (MSC) and Fishery Improvement Projects (FIPs) are two models of private fisheries governance whose presence and influence has grown significantly in recent decades [5,6]. While the MSC is a private standards-setting body, FIPs are a template for improved fisheries governance, developed over time by multiple organizations (of which Sustainable Fisheries Partnership (www.sustainablefish.org) and World Wildlife Fund (www.worldwildlife.org) are the two most prominent, with the aim to make fisheries more sustainable by providing a strategic plan for actions that should lead to a change in policy or practices [7]. Despite their remarkable growth–from a handful in 2007 to 107 in 2015 –FIPs have received limited attention in the governance literature. Studies about FIPs are largely limited to individual case studies [8–12] and a few consultancy reports [6,13,14]. Three notable exceptions include Sampson et al. [15], Cannon et al. [7], Thomas Travaille et al. [16] but these authors focus primarily on measurable outcomes, often in the water. Such information of environmental outcomes is naturally key for evaluating fishery improvement over time, and to inform the discussion on whether FIPs are a legitimate marketing strategy for sustainable seafood (based on assumptions of stock improvements). However, assessment of the governance process–in other words the strategic decision-making and resource use of a FIP–is equally paramount for understanding the process and pathways that take a FIP from initial stages of implementation to a place where key achievements are made (c.f. value chain governance [17]). Such an analysis allows for the identification of intermediate outputs that can indicate a trajectory of positive (or negative) change long before measurable outcomes in the water are actually seen. As such, it provides an empirical base for engaging with the critique levelled against FIPs for the poor performance over time [7,15].

To address this gap, this article provides a systematic analysis of the actions undertaken within FIPs to promote fisheries sustainability and the actors involved in each action, in different parts of the world, and across different fishery types. In doing so, we provide the first (known to us) global analysis of FIP governance processes with the aim of providing insights into the kinds of governance outputs generated by this mode of governance. Our findings show a diversity of strategies but also an emerging dichotomy in how FIPs develop strategies to affect change; either through engagement with policy or through practice. We discuss these findings in relation to recent theoretical developments surrounding private governance and sustainability partnerships [18–20] and reflect on both the opportunities of FIPs to contribute to sustainable fisheries governance, but also challenges that will most likely need to be addressed in order for this governance form to reach its full potential.

## 2. The rise of private governance and Fisheries Improvement Projects

A recent review of over 50 years of scientific literature highlights the evolution of fisheries governance focus over time, as well as the academic community’s corresponding shift in analytical focus [21]. This shows a preoccupation with state control in the (1950–1980); more collective governance approaches in 1960s-2000; eventual devolution of state power to user groups through co-management in the mid-1990s-2000; and increasing use of privatization mechanisms (such as individual transferable quotas and territorial use rights) from the 1990s to the present.

However, the early 1990s also saw a parallel emergence of a broader movement around fisheries and conservation in response to multiple observed fisheries governance failures by governments, in North America and elsewhere. Sutton and Wimpee [22] describe this ‘sustainable seafood movement’ as a push, by a multitude of (primarily non-state) actors, for a broadening of fisheries governance beyond public policy and for leveraging the market to build powerful incentives for ocean conservation. A key initial outcome of this movement was the establishment of the Marine Stewardship Council (MSC)–a now globally recognized private governance initiative that has received a lot of attention, acclaim and criticism [23–26]. Private governance represents an increasingly blurred delineation between the public sector (such as governments enforcing state regulation) and private (non-state) institutions (such as NGOs or markets actors making decisions about allocation of non-public resources), which historically have been viewed as having completely separate and distinct roles [19]. The establishment of the MSC was part of a larger trend of private governance for sustainability pursued also for other commodities, such as timber and coffee [27–29]. In fact, the 1990’s marked the rise of an era where private governance has become a key feature of production networks in both terrestrial and aquatic settings [20,27,30–32]. As such, they exist alongside, and often interact with, the public sector.

There are several reasons for this development, particularly in the marine realm. First, private governance emerged in an era when citizens were encouraged to use their consumer power to influence [33], and was therefore closely tied to an increasing sense of individual responsibility, coinciding with improved information availability. Second, partnerships between state and private actors were becoming more common in general, and went beyond mere interaction between these previously separate actors [34] to now combining the market-based power of lead firms with the legitimacy of states to regulate marine resources [35]. Thirdly, more and more companies saw (and still see) multiple values in pursuing sustainability, ranging from pure economic incentives (competitiveness, market access) to an increasing concern with their social license to operate [36–38].

It is in this context that FIPs came to be. Today, the most common way of defining FIPs is set out by The Conservation Alliance for Seafood Solution (CASS), an umbrella platform of major NGOs (including SFP and WWF) engaged with sustainable seafood issues and FIPs [7,39]. CASS defines FIPs as multi-stakeholder efforts to address environmental challenges in a fishery by harnessing the power of the private sector to incentivize positive changes toward sustainability in the fishery and seeking to make these changes endure through policy change [39]. Multiple organizations have adjusted and adopted FIP as a tool for improving fisheries sustainability and FIPs exist in multiple shapes and sizes. Some are industry-led, while others are championed by NGOs. They range across many different kinds of fisheries and operate in both high- and low-income settings. Although the FIP tool allows for flexibility, CASS has developed five key criteria FIPs need to draw on: 1) active participation from private actors in the supply chain (e.g., suppliers, retailers, fishing industry). Other actors involved in the FIP could be for example NGOs, governments, research organizations; 2) public commitments by participants to financially invest and make improvements to the fishery; 3) defined near-term scope of the project with a set of time bound objectives; 4) a work plan with an associated budget and deadlines. The work plan needs to be publicly available; and 5) a progress report to be able to regularly track work toward the activities and objectives defined in the work plan which also needs to be publicly available [39]. The scope of a FIP, its objectives and verification process can vary and be contextually adapted. FIPs are therefore currently divided into two types, either *Basic* FIPs which imply that the FIP focuses on one or two specific environmental issues or *Comprehensive* FIPs which means they address multiple environmental problems and often aim to enter MSC certification [39].

## 3.Methods

### 3.1 Methodological approach

Our analysis aims to understand what actions FIPs employ to affect change, what these lead to, which actors are involved, and how these actions are deployed across geographic contexts and fishery types. The paper therefore centres on a global analysis of the FIP working process (referred to from now on as the ‘FIP governance process’). We see the actions engaged in by FIPs, and reported on in publicly available progress reports, as reasonable proxies for their enactment of fisheries governance. This also forms the only currently publicly available material by which to examine the FIP governance process. Given the articulated ambition of FIPs to use supply chains as a mechanism for implementing sustainable practices, a study of how FIPs contribute to fisheries governance therefore requires systematic examination of the decisions made across supply chain actors at multiple levels, as well as the strategies behind these decisions and the management choices made to implement them [17]. Our analysis therefore proceeds in three steps. First, we compile a database of all known FIPs (as of 2016). Second, we develop a set of criteria to evaluate how assessable the FIP governance process is based on the quality of the publicly available report. Third, we develop a coding framework to analyse actions, actors involved, and outputs which we then deploy across our analysis of a global set of FIPs with publicly available reports that satisfied the assessibility criteria noted above. Below we outline each of these methodological steps in more detail.

### 3.2 Data compilation and organization

We first compiled a database of all known FIPs containing key characteristics. In the end of 2015 SFP shared a database listing 107 FIPs with the research group. The database was complemented by the researchers and ultimately contained the FIP name, main target species, FIP start date, FIP activity status, organization initiating and currently running the FIP, MSC status (if any), FIP country, geographical region, and, when information was available, reason for inactivity, among other things (for a full report of all database fields see S1 Table). For the sake of analysis, we categorized target species into broad groups of species (Crab/Lobster, Tuna, Shrimp and Others).

### 3.3. Data, sampling, and criteria for inclusion

As mentioned earlier in section 2, FIPs’ must (according to the guidelines made by CASS [39]) develop a work plan which identifies objectives and a strategic plan to research these, to officially launce the FIP. Thereafter, FIP participants must publicly post a progress report online and provide evidence of the actions completed. For all FIPs we searched for publicly available FIP reports using www.fishsource.com and www.google.com. Reports were downloaded and read during October 2015 to February 2016. At the time of data collection (Oct 2015-Feb 2016) there was no standardized FIP reporting template; or common webpage for all FIPs’ progress reports. However, reports often followed the format advocated by either Sustainable Fisheries Partnership (SFP) [40] or World Wildlife Fund (WWF) [41]. Progress reports were either *i)* a FIP Progress Update Table which corresponds to five different FIP stages, together with an explanatory text of the progress update or *ii)* a FIP tracking sheet, based on the MSC Benchmarking and Tracking tool in which activities are reported against the MSC Fisheries Standard [42]. Even though FIPs are not related to MSC it is common practice for many FIPs to use the MSC Benchmarking and Tracking tool, as a means to report their progress. These progress reports (from here on referred to as ‘FIP reports’) constitute the raw data of this study (see S1 Appendix for more details). Since 2016, after the data collection for this study, Fisheryprogress.org was established which serves as a consolidated source for information on FIPs [43].

Next we rated reports based on how assessable they were. In other words, we assessed the degree to which reported information allowed us to code for actions and associated outputs (see section 3.4 on coding framework). Reports were classified as 1) ‘assessable’ if they provided sufficient information to allow us to evaluate FIP actions and outputs using our coding framework; 2) ‘not assessable due to weak report’ if they did not provide sufficient information for any analysis; and 3) ‘not assessable due to inactivity’ if the FIP has been officially discontinued (see S1 Appendix for details on criteria and classification). Of the 107 FIPs in the overall database, 56 FIPs were assessable.

### 3.4 Analytical framework and coding

The third step in our analysis required the development of a coding framework. This was primarily a deductive framework, with the overarching structure was thus largely informed by our desire to capture the actions FIPs employ to affect change, what these led to, and which actors were involved. This led to a code hierarchy with actions/actors/outputs as the top level hierarchy, under which we developed further subcodes. However, the subcodes were developed through the reading of 10 reports and doing thematic coding (constrained by the three top level codes noted above). In an iterative process between reading and code development we thus eventually arrived at the codebook. A full description of the framework, and all codes can be found in S2 Appendix.

Outputs are direct evidence of the types of governance actions FIPs undertake and they are defined here as distinct achievements directly related (and to some degree traceable) to specific actions coded for in the FIP, including changes in both policy and practices. Policy outputs are related to actions that aim to achieve a change related to policy and/or regulation, by targeting policy actors, arenas or processes. Examples include engaging in processes to change laws, fishing regulations, quotas, or monitoring and actions that aim to improve governance. Practice outputs are related to actions aimed at achieving a change in practices (not relating to government regulation), by targeting non-governmental actors, arenas, or processes (e.g., NGOs, industry). Examples include initiation of traceability schemes–a process which does not always necessitate government enforcement or involvement. Non-government enforced gear changes or changed fishing practices are also included in this category. Often these practices are used in place of government intervention to affect change in a system.

The final codebook includes 12 main action codes (24 sub codes); four main output codes (11 sub codes); and eight actor codes (each listed in Tables A, B and C in S2 Appendix). Each action noted in a FIP report was coded according to an action type. A FIP can have multiple action types at the same time. Only actions reported in the FIP reports were coded, thus we did not include background information or FIP objectives. Only activities listed after the FIP was launched were coded.

Each reported output was coded. Only outputs that very likely happened because of the involvement of the FIP were coded for and included in analysis, while we did not, for example, include new laws that lay outside the direct influence of the FIP, and were likely to have happened regardless (even if sometimes reported in the progress report of a FIP). If the FIP was documented as central to the law being passed, or if the FIP specifically crafted content for a law, then this was included. The coding applied will therefore recognize the “bridging” or “catalyzing” role of FIPs but may underrepresent the role of some FIPs in actual policy creation.

For all strategies and outputs coded, we aimed to identify which actors were involved. Actors were divided into seven categories (Table B in S2 Appendix). If the specific actor involved was not identifiable, but it was clear that some/or all FIP participants were involved in an action, all FIP participants were coded as participating, thus potentially overestimating actor participation in some instances. If no actors could be reliably linked to the action it was coded as ‘no data’.

#### 3.4.1 Coding procedure

After the codebook was developed it was tested and refined in multiple steps. First, three researchers coded the same ten FIP reports independently applying the framework. Discrepancies between coding was noted and any ambiguities in interpretation discussed. Based on this the codebook was refined; some categories were grouped together and others were removed. In the second testing round, ten additional (not coded before) reports were coded by the same three researchers. A similar procedure of discussion and refinement too reduce discrepancies in interpretation took place and the codebook was once again refined. After this round the codebook was shared with SFP staff with long-term experience in dealing with FIPs. The expert feedback on code categories was evaluated, codes were again revised and one final round of coding of ten reports was done to assess inter-coder agreement of the final codebook using Krippendorffs’ Alpha, applicable for any number of coders [44]. With Alpha = 61, and 73% percentage agreement, specific areas of inter-coder discrepancies were identified and aligned, after which all FIP reports were coded using the finalized codebook. All coding was done using MAXQDA [45]. Coded segments were then exported to Excel spreadsheets for further statistical analysis and exploration (Microsoft Office 2016).

## 4. Results

### 4.1 Global distribution of Fishery Improvement Projects

**Fig 1:**
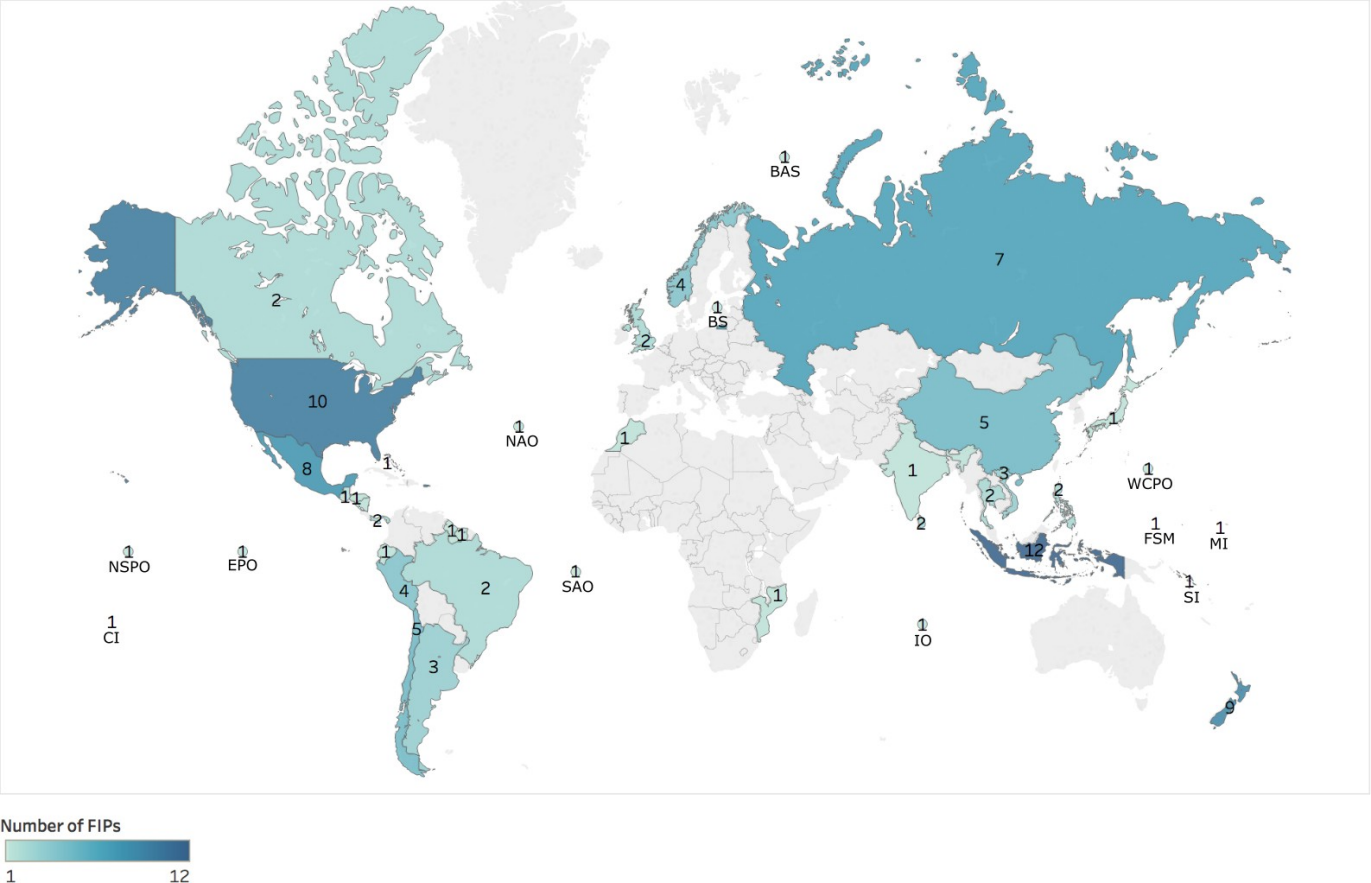
Worldwide distribution of Fishery Improvement Projects (FIPs) in 2015. Numbers within countries represent the number of FIPs per country. Numbers in oceans represent the region of multinational FIPs. Letters represent the names of ocean regions or islands nations. Darker colors of countries indicate larger number of FIPs. (BAS: Barents Sea; BS: Baltic Sea; CI = Cook Islands; EPO: Eastern Pacific Ocean; FSM: Federated States of Micronesia; IO: Indian Ocean; MI: Marshall Islands; NAO: North Atlantic Ocean; NSPO: North South Pacific Ocean; SAO: South Atlantic Ocean; SI: Solomon Islands; WCPO: Western Central Pacific Ocean). Figure made using Tableau Software [46].

FIP presence has grown, with the number of FIPs steadily rising from four in 2007, to 57 in 2011, and 107 in 2015. As of 2015, Asia and South America had the largest number of initiated FIPs, followed by Europe, Central America and North America (Fig 1), although data published by the California Environmental Associates indicates an increase in FIPs in North America and Oceania since 2016 [14]. While the total number of FIPs initiated has increased over time, the FIP process has been discontinued in several of these fisheries.

### 4.2 Regional differences in FIP presence

**Fig 2:**
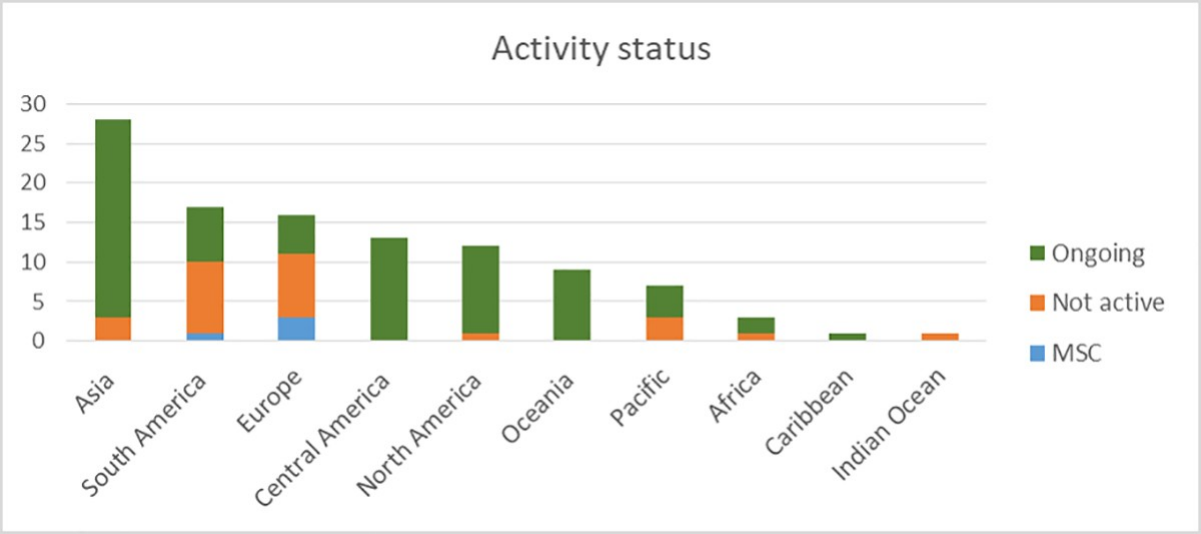
Activity status (as of 2016) of FIPs in different geographic regions.

Regional differences in FIP presence was observed and linked to fishery type (Fig 2). In South America, the number of fisheries where a FIP process was initiated is high; however, 53% of these FIPs were not active as of 2016. Similarly, half of the European FIPs were not active, while three (around 20%) have MSC certification.

In South America the discontinued FIPs were all run by the same NGO, and in Europe four FIPs were suspended because some industry actors were not sufficiently committed or relationships changed. Across other regions, the reasons for suspension are not always documented but include lack of industry commitment, or discontinuation after the scoping stage. Small pelagics and whitefish represented 35% and 31%, respectively, of all inactive FIPs, while no crab, lobster or shrimp fisheries had been discontinued as of 2015. Several of the small pelagic fisheries (as well as the North Sea sand eel fishery) were suspended as a result of insufficient interest from supply chain actors in promoting improvements in these fisheries due to the small contribution to overall volumes in their supply chain.

### 4.3. FIP actions employed to change policies or practices

The most commonly observed action employed across all fishery types and across all regions were data collection and dialogues initiated to discuss data collection needs, collection approaches and responsibilities (see Table 1 for the most common actions, also S1 Fig). Looking across all reports commonalities in types of actions were primarily evident within fishery types, not across regions. As such we limit our detailed presentation of results to the former.

**Table 1:** Top three most common actions reported, and outputs observed, in FIPs across various fishery types. () in column headings indicate total number of FIPs analyzed. The last row summarizes the type of organization leading FIPs in each type of fishery. In cases where multiple action types are listed under 1,2 or 3, these actions were all ranked the same (in terms of how common they were) across FIPs of that fishery type. (%) reported after each action (1 to 3) indicate the percentage of FIPs in which this action type was represented as one of the topmost reported actions. Taking shrimp fisheries as an example, 60% of all shrimp FIPs engaged in the four actions listed as the most common (i.e. these were equally common in terms frequency of mention), while 30% had the second most common, and only 20% reported basic dialogues, which was the 3^rd^ most common based on frequency of mention.

	Crab/lobster (13)	Shrimp (10)	Tuna (13)*	Others (20)
**Most common actions**	1.Data collection: 92% 2.Data dialogue; Engaged policy dialogue; Education: 54% 3.Basic dialogue practice; Basic dialogue policy: 46%	1.Basic dialogue policy; Engaged dialogue practice; Data collection; Data dialogue: 60% 2. Education: 30% 3. Basic dialogue practice: 20%	1.Basic dialogue policy; Engaged dialogue practice: 92% 2. Data collection; Data dialogue: 69% 3. Basic dialogue practice: 62%	1. Data dialogue: 85% 2. Data collection: 70% 3: Basic dialogue policy: 60%
**Most common outputs**	***Policy outputs 62%*** Management plan 23% New management body 23% Limited entry 15%	***Policy output 40%*** Management plan 20% Compliance 10% New management body 10%	***Policy outputs 15%*** Management plan 8% New management body 8%	***Policy outputs 20%*** Quotas 10% Management plan 5% Limited entry 5% New management group 5% Fishers support program 5%
	***Practice output 23%*** Traceability schemes 23% Fishers’ association 8%	***Practice output 60%*** Observer programs 40% Traceability schemes 20% Gear change/restriction 20%	***Practice output 31%*** Gear change/restrictions 23% Traceability schemes 15% Observer programs 8% Fishers’ association 8%	***Practice output 40%*** Traceability schemes 15% Gear change/restrictions 15% Observer programs 5% Bycatch utilization 5% Fishing stop 5%
**Actor running FIP**	NGO 46% Industry 38% Fishers 8% Research org 8%	NGO 70% Industry 30%	Industry 54% NGO 15% Consultancy 8% NGO/industry 8% Fishers 8%	NGO 65% Industry 20% Fishers 10% NGO/industry 5%

*counted for panama tuna and mahi mahi in the tuna category

Reports show that FIPs often have a strategic focus on changing fisheries governance in the government itself. Dialogues with policy stakeholders was thus another action employed across all FIPs. However, only crab and lobster fisheries were engaging in deeper and more sustained conversations in this domain when looking at the three most common actions. These same fisheries also show a larger percentage of policy related outputs (Table 1). All other fishery types report only basic policy dialogues, which means that they are merely exploring new ideas, or engaging in one-way communications by sending letters or requests to policy actors urging policy changes, without any documented evidence of deeper discussions or sustained dialogue.

In addition to influencing public policy, FIPs also aim to change fishery practices. Many FIPs that did not report more than basic policy dialogues instead engaged more in dialogues around practice. FIPs engaging in practice dialogues included shrimp and tuna fisheries, as well as crab and lobster fisheries (Table 1). Both shrimp and tuna FIPs report sustained engagement in processes to test and implement changes to fishery practices. These practices include, but were not limited to, experiential education (such as gear training, logbooks, handling programs training), observer programs, and traceability improvements. Crab and lobster fisheries, on the other hand, reported lower levels of engagement around practice (‘basic practice dialogue’).

Lastly, education was the third most common action employed, but reported primarily by FIPs involved in shrimp and crab/lobster harvesting. Education here refers to more conventional education such as dissemination of information regarding management regulations, ecological data or sustainability issues, while experiential education in the form of gear training, logbooks, handling programs is encompassed in the engaged practice dialogue examined above. As such, tuna FIPs, which report significant amounts of such experiential education through their pilots, do not show a high rate of conventional education strategies in Table 1. Shrimp FIPs focus primarily on educating fishers about regulations and bycatch reduction (such as the use of Turtle Excluder Devices (TEDs)), while crab and lobster FIPs report educating fishers about regulations and the existence of the FIP through use of radio and information videos respectively, as well as distributing information documents on regulations and best practice, and cigarette lighters to promote measuring of crab size.

### 4.4 Common FIP outputs

Table 1 outlines the most common FIP outputs. A management plan and a new management body are the most common policy outputs, indicating the desire by many FIPs to contribute to changing fisheries governance. Often these new bodies are multi-stakeholder groups, including industry, NGOs, government, and fishers. These management bodies generally aim to foster more collaboration between stakeholders around management issues in the fishery, and appear to help FIPs institutionalize their work. One example of a new management group is the Longline Fisheries Commission in Panama, which is a result of the Panama Mahi Mahi and Yellowfin tuna FIP. The commission includes representatives of the fisheries authority (ARAP), fishermen, mahi-mahi and tuna exporters, operators of longliners, fisheries researchers, and NGOs. It aims to create a participatory Management Body, able to make decisions in order to achieve a sustainable status for longline fisheries in Panama.

As noted above, many FIPs are also trying to influence fishery practices. Traceability schemes are a practice output that ranked high across all fisheries. As traceability is a key step in achieving certification and essential in improving transparency in fisheries supply chains globally this is not surprising. Examples of different types of traceability schemes reported across the FIPs include control documents, policies on sourcing, and both voluntary and policy mandated controls of gears.

Observer programs stand out as a commonly reported output in shrimp and tuna FIPs. The offshore nature of these fisheries, and the importance of reducing vulnerability to transshipment and bycatch issues in order to align with traceability priorities [47–49] may explain this pattern. Finally, all fishery types, except crab and lobster, report practice outputs in the form of gear restrictions and/or change. Crab and lobster FIPs instead use procurement specifications, and sourcing restriction on undersized or berried females.

### 4.5 FIP actors and the actions they employ

**Fig 3:**
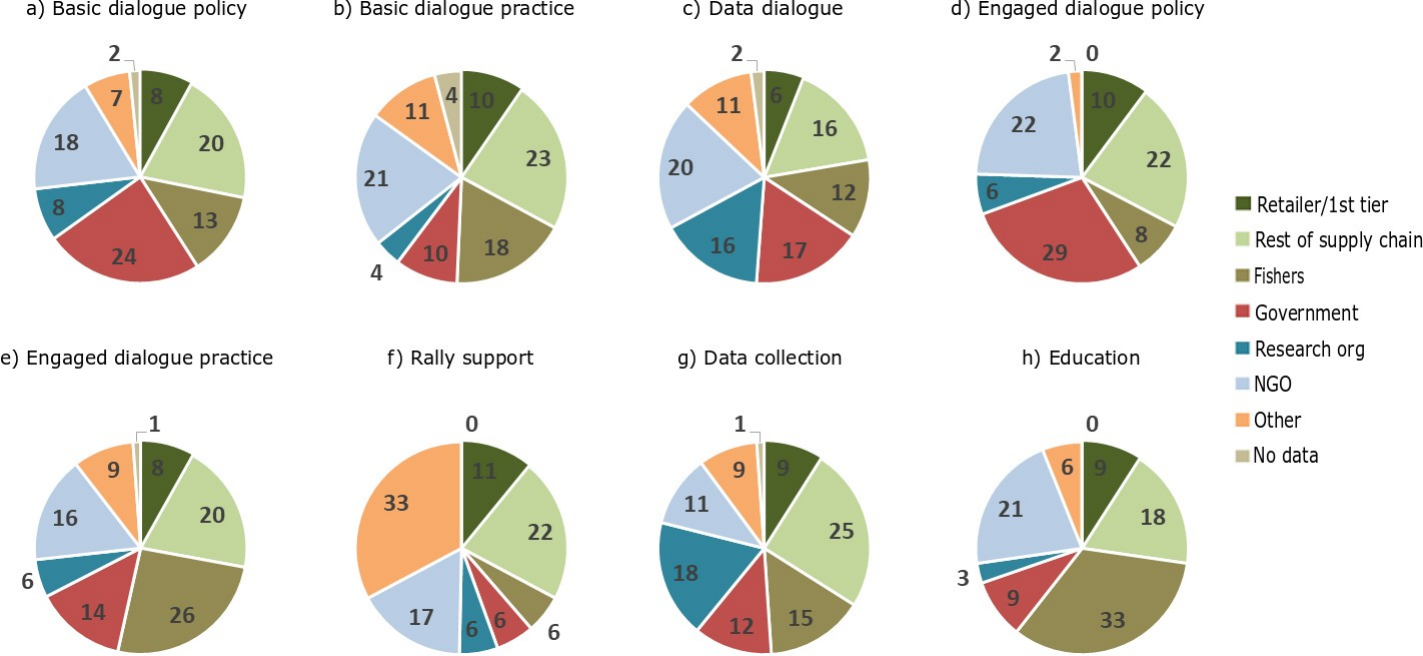
Representation of actors engaged in FIP actions across all fisheries. Pie charts show the engagement of actors in action types aggregated across all fisheries. For each of the eight action types (i.e. each individual pie chart), the engagement of a particular actor type is shown as a percentage of all coded records of actor-action combinations, across all fisheries. For an analysis of actor-action combination in specific fishery types, see S2 Table. ‘Others’ represents a mix of other actor types, but consultants are overrepresented in this category. ‘No data’ represents reported actions where no specific actors were identified as contributing.

While the previous section outlined the most common types of actions engaged in by FIPs, this section examines which types of actors were commonly involved in these actions. Fig 3 shows the association between various types of FIP actors and reported FIP actions. In other words, who is involved in what actions. This figure shows that FIPs engage actors from all levels in the supply chain, though their roles in specific actions are often specific. If we examine the involvement of actors across all fishery types (Fig 3) two clear patterns emerge. Collection of, and dialogue around, data is a domain where involvement of research organizations is significant, regardless of fishery type. It is also clear that supply chain actors (excluding retailers and 1^st^ tier suppliers) are not strongly associated with any particular action but consistently involved across all, with the exception of shrimp fishery FIPs. A more detailed look at individual fisheries shows that in the shrimp FIPs, for example, retailers and 1^st^ tier suppliers have played a more pronounced role particularly in rallying financial support for the FIP, but also in practice dialogues, in educational efforts to distribute educational material to captains, and through data collection related to bycatch (S2 Table).

The unique and different roles that governments and industry actors appear to take within FIPs are clear in our analysis. Governments engage primarily in policy dialogues. Fisheries arguably require data collection to improve governance, yet government actors are underrepresented in data collection efforts across all FIPs.

Across all fishery types, fishers participate in education and changed practice efforts. Many of the pilot projects and both traditional and experiential education initiatives conducted through FIPs are done in collaboration with fishers, explaining this pattern. However, apart from logbook trials, fishers are rarely reported to be involved in data collection or policy dialogue, which suggests they are not directly involved in conversations around new regulations.

NGO involvement appears to be context dependent, with high involvement across multiple types of actions in crab and lobster, and others fisheries, while in shrimp fisheries engagement is mainly in dialogues around practice and policy, and in tuna fisheries education and rally support are more common (S2 Table).

## 5. Discussion

### 5.1 Emerging patterns of FIP actions and governance outputs

Our analysis highlights a number of interesting patterns in the presence, actions, and outputs of FIPs around the world. First, the types of observed actions to improve sustainability differ across fishery types. Crab and lobster FIPs report deeper policy engagement, while shrimp and tuna FIPs generally report stronger engagement with practice. For example, in the blue swimming crab FIPs in Southeast Asia, the industry is working closely with governments to develop guidelines for best practices and improved regulations. Indonesian government officials have travelled to the US to meet with the National Fisheries Institute Crab Council to learn more about fishery management. Since Asian crab fisheries have relatively recently entered the global market and have previously not been as heavily regulated as some other fisheries, it is possible that the entrance on a global scene has spurred a closer initial dialogue process between industry and government to put regulations in place and stem indications of overfishing.

The stronger engagement around practice across tuna and shrimp FIPs may result from the fact that governance discussions of tuna often take place at the level of regional fisheries management organizations (which are often not accessible to FIP actors), hence engagement with policy actors is less accessible. Tuna fisheries do stand out in their heavy involvement of industry actors as drivers of FIPs. In the case of shrimp FIPs, many of these were primarily initiated to deal with issues related to bycatch, a problem closely linked with fishing practices and gear types. Efforts to find gear and practice based solutions were therefore most likely a more direct way of dealing with this issue than working via policy development, and thus explains our observed pattern.

One of the most common actions reported across all FIPs is data collection, and dialogues relating to it. This is not surprising, as data deficiency is one key reason for initiation of many FIPs, and data availability is a key priority for the MSC benchmarking process which many (but not all) FIPs follow [42]. What is notable though, is that data collection is primarily focused on only biological data. Furthermore, most FIPs collect data only on the species in focus in the FIP even though it is clear that in many fisheries, particularly in developing countries, fishermen engage in the extraction of multiple species [12,50,51]. Also notable is that no FIP reported data collection on fishers’ behavior. Data collection on single species impedes an ecosystem approach to the governance of these fisheries, which is commonly recommended [52,53] and the lack of socio-economic data such as fishing behavior precludes the possibility for assessing behavioral change over time. Data collection on additional social impact and aspects, including how the fisheries can offer flexibility, viability, and security to the fishers and the community, is generally seen as equally relevant for fisheries sustainability (see Van Holt et al. [54] for an example of how FIPs are considering to incorporate social issues into FIPs), but is also notably absent from the data collection reported by FIPs reviewed here.

Finally, how assessable and comparable FIPs are over time is critical to evaluating progress. It is therefore noteworthy that just under half of the FIPs collated for analysis were not assessable according to our criteria (Fig A in S1 Appendix). As noted in the introduction, examining environmental outcomes is key to evaluating fishery sustainability over time (see e.g., [7,15] but without an assessment of the governance process, strategic decision-making and resource use of a FIP, our understanding of their pathways to success or failure will be poorly captured. This will limit our understanding of how FIPs operate and hamper learning that could otherwise improve the FIP governance process and be capitalized on by future FIPs.

### 5.2 Complementarity and mutual reinforcement in fisheries governance—a preliminary heuristic for understanding FIP contribution to fisheries governance

The rapid growth of FIPs suggests these improvement initiatives are becoming a feature of the global fisheries governance landscape that deserves attention. It is therefore important to examine the potential contribution of this evolving private governance mode in relation to theories of influence emerging from the broader private governance literature (e.g., [19,27,28]) The format of FIPs–with collaboration across multiple actor types, ranging from NGOs, to research organizations, private companies, local extractors, and sometimes also government bodies–places them squarely within what is typically defined as private governance. Namely, governance driven by various types of non-state actors, who interact to produce institutional arrangements that structure and direct actors’ behavior in a particular domain [4]. Some have also referred to this mode of working as sustainability partnerships [18,19], described as “*collaborative arrangements in which actors from two or more spheres of society (state*, *market and civil society) are involved in a non-hierarchical process through which these actors strive for a sustainable goal*” (p.2 in [18]). Gulbrandsen [29] and Groenveld et al. [19] show how such state and private sector partnerships can be both complementary, but also mutually reinforcing of the legitimacy of state regulation. Building on these ideas, and anchoring our discussion in the findings of our analysis, we propose that complementarity and reinforcement could form the basis for a simple heuristic by which to begin to classify and understand the contribution of FIPs to fisheries governance. We note however, that in order to fully evaluate the contribution of FIPs against this heuristic would require development of more stringent criteria for what should be considered complementary and reinforcing dynamics, and an application of this across FIPs. But as a first step, we develop our ideas below, and draw on examples from our analysis to support them.

#### 5.2.1 FIPs as complementary to government strategies

FIPs reviewed here appear to engage either to influence policy, with the involvement of governments; or to change practices, with the engagement of supply chain actors. This division of labor is logical given limited resources available, and may also be a reflection of the complementary role that governments, private actors and civil society have been noted to play for governance of fisheries and other resources (e.g., [19,28,55]). When it comes to changing practices, many of the activities happening within FIPs studied here are undertaken as collaborative efforts between the capture sector and various supply chain actors, without the involvement of government. This makes sense as market actors, through their engagement with producers, are well placed to develop and help implement new technologies or practices. The complementary role of non-state actors developing novel practices, could therefore be seen as an important source of innovation, but one that can and should be complemented by enabling institutions [56,57]. In order for novel practices to contribute to enduring change for sustainability, they often require institutional support to enhance them and allow them to scale up [58]. In many cases, such support needs to be developed, through crafting of new regulations or adjustment of policy mandates within government bodies, to mention only a few examples. This important role of ‘enabling’ institutions and legislation in supporting and enhancing various transition processes towards sustainability has been documented in multiple cases [27,56,57]. Drawing on our sample of FIPs, an example of how this type of complementary actions can play out can be seen in the blue swimming crab FIP in the Philippines. In this case, the lead firms of the value chain, importing processing companies from the US, implemented sourcing policies, for example a restriction on purchasing egg-bearing crabs, with the aim to improve the crab population. The FIP also engaged in dialogues with policy makers through stakeholder consultations and later some measures from the private sourcing policies were included in the blue swimming crab Management Plan that was adopted by the Philippine government. Another example is the case of the South Java Handline yellowfin tuna FIP in Indonesia. There, the industry-led FIP ran a Circle Hook Program in which they distributed free circle hooks and had training sessions with fishers to encourage them to change from the traditional used hook in order to reduce bycatch. At the same time the FIP supported the development of national regulations for bycatch mitigation measures and harvest control rules for the fishery. The lack of reported government involvement in many FIP actions related to practice is therefore noteworthy, and suggests that in order for these FIPs to be able to fully realize their potential for complementing existing governance structures and process (such as fisheries regulation) more focus needs to be directed at better aligning policy and practice in the strategic actions taken by FIPs.

#### 5.2.2 Mutual reinforcement of state regulation

Traceability is a good example of how FIPs can, and are, mutually reinforcing existing regulation. Where data on where, and how, a product was caught or processed is poor or non-existent, enforcement of regulation is necessarily undermined. This is increasingly recognized in the fisheries sector, and has sailed up on the sustainability agenda as one of the key sustainability challenges to overcome [59]. Given the imperative to know the origins of any product, supply chain efforts to improve traceability can therefore substantially reinforce or facilitate the enactment by government of state regulation [60]. We identified several examples of how traceability measures were used in the FIPs. One example is the Gulf of California Industrial Shrimp FIP where the fishery experienced problems with compliance and regulatory enforcement. To improve the problems the FIP engaged in a traceability program and demanded that all FIP participants (importers) sign a control document, a verifiable mechanism for documentation of the supply chain. With this control document the FIP aimed to exclude vessels that were violating Mexican federal fishing regulations (e.g., fishing in restricted areas). The FIP developed a third party audit system in which vessels under the control document were audited to ensure compliance with national regulations. In the case of the Brazilian Lobster FIP, a traceability scheme was implemented to promote legally caught lobster. The scheme was linked to a market brand and aimed to obtain market recognition for fishers that complied with fishing gear regulation and only used traps. Another example is the Longline Tuna FIP in the Federated States of Micronesia, where the FIP introduced radio frequency identification tags on frozen tuna to provide full traceability from the vessel to the end of the supply chain. The FIP had before that implemented a policy banning the retention of sharks and fishing gears targeting sharks, to comply with domestic regulation. The multiple FIP traceability schemes identified through our analysis of FIPs can therefore be seen as a step towards potentially reinforcing both environmental, social and public health related regulation through collection of information on stocks fished, gear used, labour rights, and hygiene processes at various points in the supply chain.

Traceability is heavily reliant on good data, and data collection is a ubiquitous action across reviewed FIPs. This speaks in favor of FIPs filling another important ‘reinforcing’ role in regions of limited government capacity, were stock status is often unclear as a result of limited monitoring capacity (c.f. [15,61]). Through the involvement of multiple segments of the seafood supply chain in the FIP, data collection could potentially be more easily captured in a way that facilitates sharing across supply chain segments and also abides by the emerging standards around so called ‘key data elements’ being developed by coalitions of leading seafood brands (see e.g., Global Dialogue on Seafood Traceability: https://traceability-dialogue.org/). However, it is equally important that governments do not rely singlehandedly on private actors to provide one of the most basic building blocks of well-functioning governance; high quality data. As Groenveld et al [19] note, “*Despite the added value of private governance*, *and the goodwill of those involved*, *it cannot guarantee that policy objectives in ocean and coastal management will be attained*. *An active role for governments will therefore remain warranted*” (p.19). In other words, there is a risk that governance vulnerability (through low institutional capacity) is perpetuated if the responsibility for key governance functions remain unarticulated or outsourced to private initiatives. Thus data collection should not be seen as a ‘complementary role’ played by FIPs, but FIPs can support and reinforce this important task. It is also worth reflecting on the fact that private governance could also be critiqued for potentially setting a system on a path dependency where too much influence is handed to private actors possibly hampering the ability of government and fishers to reverse this and regain control of norm-setting and regulation[62,63]. However, this is a broader topic, well beyond the scope of this paper.

### 5.3 Some critical reflections on FIPs and their potential to contribute to fisheries governance

While the growth in numbers and diversity of FIPs worldwide is impressive, it is worth briefly noting some of the key potential challenges that our analysis brings to the fore, and which should be further engaged with by FIP actors and promoters to fully deliver on their potential to promote fisheries sustainability. One such challenge relates to the current and noted lack of data collection on multiple species, as well as fishers’ (or other market actors’) behavior, which precludes both more ecosystem based management decisions (e.g., [64,65]) but also an assessment of behavioral change over time. The latter is a missed opportunity for all actors involved to understand a key mechanism for transforming towards sustainability, which has been highlighted by behavioral scholars in multiple disciplines [66–68].

Another noted challenge relating to learning and improvement over time is the lack of standardized reporting. Here we do see an encouraging trend towards efforts at standardizing reporting and also capturing more of the social and governance processes behind FIP outcomes in the revised reporting system coordinated by Fisheryprogress.org, a web-based FIP progress tracking tool supported by multiple organizations and currently led by FishChoice (www.fishchoice.com). However, learning also requires understanding of failures and their antecedents, which were rarely reported in the material reviewed for this study. Finding ways to also capture informal or even unfruitful strategies, and the contexts in which they were pursued, means valuable insights may be gained for the future.

A third challenge emerging from our analysis is the notable lack of participation of fishers’ in many of the FIPs analyzed. Only 25% of FIPs examined report engaging both fishers and retailers or 1st tier suppliers in their reported activities, and when they do, it is primarily related to various training programs involving fishers, ranging from appropriate gear use, to log books, monitoring, and handling practices. This is positive, and in line with conclusions made by e.g., Tolentino-Zondervan and colleagues [12] who note that FIPs must foster fishers’ capabilities in addition to higher ex-vessel fish prices. However, only 7% of FIPs in our study included fishers as one of the FIP lead actors, and there is a notable lack of articulated strategies for how to achieve fisher capacity building in most FIPs reporting, nor is socio-economic data that could improve understanding of social impact of changed practices generally collected (as noted above). The lack of FIP focus on fisher participation may be the result of lead-firms, such as retailers, still focusing their attention on 1st or 2nd tier suppliers. This signals a challenge for FIPs, as it is increasingly recognized that many of the perceived changes needed will have to happen by interacting with fishers on the ground (see Van Holt & Weisman [55]), and small-scale producer participation can be key for long-term endurance and local buy-in from extractors [69]. Furthermore, a narrow focus on coordination only between lead-firms and their immediate suppliers underutilizes the advantage of conceptualizing economic relations in terms of chains, and risks undermining the legitimacy of both policy and practice outputs with key producers [31,70–72].

Finally, it is also important to openly reflect on the inherent weaknesses of the FIP model of influence, which stems in part from the organizational model itself. As a mode of private governance, FIPs generally rely heavily on supply chains as a mechanism for implementing more environmentally sustainable practices [73]. While this reliance on supply chain involvement is a key strength of FIPs, and a means by which they can contribute meaningfully to governance (as noted above), it also entails some potential constraints. For one, it means that actions that cannot easily be tied to some market advantage, like a price premium or increased potential market share, may not be as easily engaged. Such critiques have been previously voiced in relation to eco-labels and MSC [74,75]. But there are also other limitations to FIP influence that can potentially be linked to the supply chain focus and lead-firm governance [17]. We elaborate on two examples below.

The first relates to the geographical contexts in which FIPs are likely to be able to be able to have a strong role in promoting fisheries sustainability. Our analysis shows that while the number of FIPs has been steadily increasing, most new initiatives have emerged in Asia, Central America and South America, and much fewer in Africa and the Pacific. The reason for this cannot be fully discerned from our data, but a look at the historical development of the retail engagement with sustainable seafood sourcing shows that large retailers have traditionally been key in driving the uptake of the MSC certification through commitments to source only certified wild captured fish[32,76]. Similarly, the retail sector has been key in the spread of the FIP model, by using pressure on 1^st^ and 2^nd^ tier suppliers to force sustainability concerns and demands up-stream in the value chain. Big retailers tend to market, and thus be interested in, in species that are traded at a global scale, and can be sourced in large homogenous volumes, such as whitefish, shrimp, crab and lobster. Notably, these are all species currently emanating primarily from fisheries in Asia and Latin America. We therefore argue that it is plausible that a reason why regions and fisheries targeting species primarily consumed in domestic or regional markets (see e.g., East Africa [77]) are not well represented among FIPs to date. It also suggests that FIPs, as a fisheries governance tool heavily reliant on lead-firm governance, may be limited in its ability to tackle sustainability in fisheries without large export markets.

A closely related issue is the fact that in some cases a particular fish stock contributes only marginally to overall supply of powerful supply chain actors, or it may be highly substitutable. Being substitutable means the commodity can be substituted by another. In a fisheries context this often happens by value chain actors (such as processors or retailers) either substituting with the same species but from a different population/stock, or in the case of products where the actual species is less important (such as whitefish in fish sticks), one species can be substituted by another species entirely (e.g., cod being replaced by haddock) (see e.g., Crona et al. [78]). This often goes unobserved by consumers, but high substitutability of certain types of fisheries commodities can undermine the usefulness of FIPs as a governance improvement tool in these cases. In other words, if pressure by lead firms to change practices is reacted to by switching to a different product or fishery instead of staying in a fishery and trying to improve it, the FIP model will not work. Our findings show signs of such dynamics, and indicate that small contribution of a specific FIP fishery to overall supply chain volumes for a particular species was cited as a reason for suspension.

## 6. Concluding remarks

We have systematically reviewed FIP modes of operation, and what FIPs are achieving in terms of policy and practice outputs. The analysis provides a global overview of geographic patterns of FIP establishment, and of common patterns of deployed strategies and outputs across fishery types. We have also highlighted the most likely challenges and opportunities for this private governance mode in the future. While providing the first global analysis of the inner workings of FIPs we recognize the limitations in our analysis which stems in part from the reliance on only publically reported data. Nonetheless, we hope the analysis can spark further interest in this topic and inform an empirically grounded discussion on the fisheries governance potential of these improvement projects, while also serving as a baseline to evaluate evolution of FIPs in the future.

## Supporting information

S1 Appendix Sampling, data organization, and criteria for inclusion. (PDF)

S2 Appendix Codebook. Codebook for examining actions, actors, and outputs in Fishery Improvement Project reports as a means to assess FIP governance.
(PDF)

S1 Dataset (XLSX)

S1 Fig Actions related to data dialogues and data collection reported across regions. (PDF)

S1 Table Metadata showing database fields available. (PDF)

S2 Table Cross-tabulation of FIP actor type by action. (PDF)
