# Supplementary material for: Fishery Improvement Projects as a governance tool for fisheries sustainability: A global comparative analysis
Source: PLoS One. 2019 Oct 1;14(10):e0223054. doi: 10.1371/journal.pone.0223054 (PMC6773218; doi:10.1371/journal.pone.0223054)
Supplement: S1 Appendix — (PDF) [file pone.0223054.s001.pdf]

# S1 Appendix. Sampling, data organization, and criteria for inclusion

FIP reports were downloaded during October 2015 to February 2016 using www.fishsource.com and www.google.com. FishSource generally provide a link to an external website used for the reporting of the FIP. In cases where the link from FishSource did not work, we used www.google.com to cross-check if there were other websites than the one on FishSource that included reporting. The report was then either downloaded (if possible) or copied and pasted in to a word document (if the text was only available on the website).

We did not include other sources, for example meeting minutes or other external sources that could have given more information about the projects. We only focused on the main compulsory reporting and not external material to be able to compare different FIPs, and not have more information for some FIPs and not others. All FIP reports included in the study were written in English.

## Criteria for inclusion in analysis

We only included FIPs with the status active (ongoing) or MSC certified. We excluded non-active FIPs. FIP reports were classified as 1) assessable [i.e. those that provided sufficient information to allow us to evaluate FIP actions and outputs using our codebook], 2) non-assessable due to weak reports or non-reports [i.e. those that did not provide sufficient information for analysis using our codebook] and 3) non-assessable due to inactivity (Table A). Only FIPs in Category 1 'assessable' were analyzed. See S2 Appendix for details about the codebook.

Please note that FIPs listed as inactive here are not active under the Fisheries Improvement Project label, but may still work with sustainability improvements under other frameworks. One example is the International Seafood Sustainability Foundation (ISSF) tuna fisheries projects, which are no longer defined as FIPs but still ongoing.

**Table A.** Summary of assessable/non-assessable FIPs

| FIP report categories | Number of FIPs |
|---|---|
| 1. Assessable | 56 |
| 2. Non-assessable due to weak report | 25 |
| 3. Non-assessable due to inactivity | 26 |
| Total | 107 |

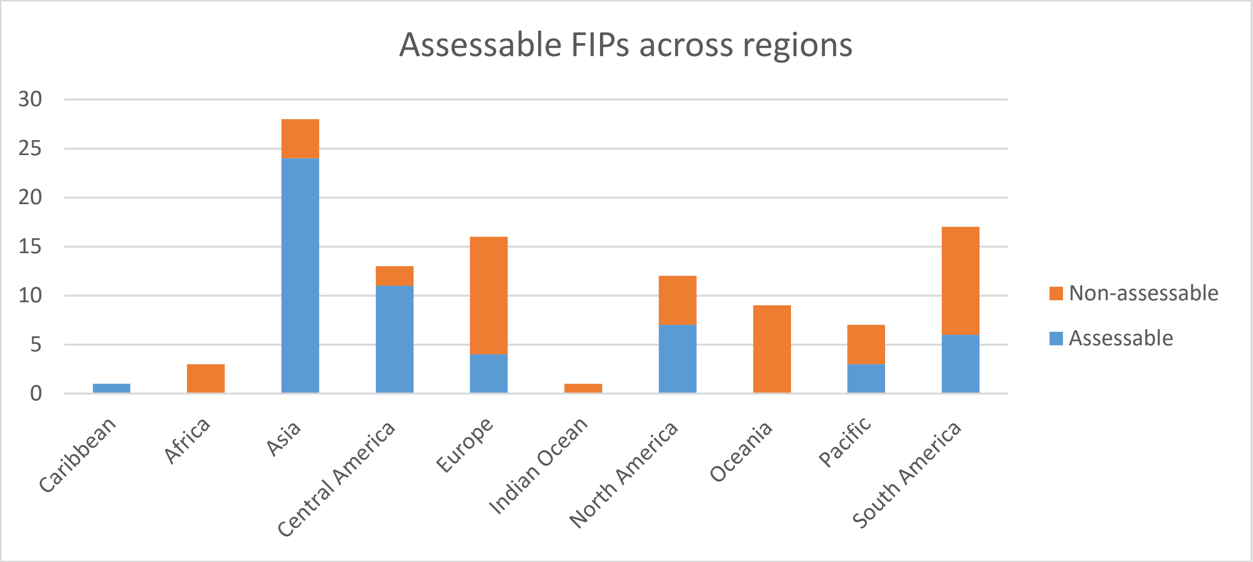


**Fig A**. The graph shows the number of assessable and non-assessable FIPs (based on quality of reports or inactivity) of FIPs in different geographic regions.
