# Supplementary material for: Fishery Improvement Projects as a governance tool for fisheries sustainability: A global comparative analysis
Source: PLoS One. 2019 Oct 1;14(10):e0223054. doi: 10.1371/journal.pone.0223054 (PMC6773218; doi:10.1371/journal.pone.0223054)
Supplement: S2 Appendix — Codebook for examining actions, actors, and outputs in Fishery Improvement Project reports as a means to assess FIP governance. (PDF) [file pone.0223054.s002.pdf]

S2 Appendix. Codebook for examining actions, actors, and outputs in Fishery Improvement Project reports as a means to assess FIP governance

In order to understand how FIPs enact governance through various actions, we developed a codebook for analysis of which actions – undertaken by which actors – appear to lead to specific outputs in FIPs. Governance efforts by FIPs in this study are understood as those actions and outputs that have been reported in Fishery Improvement Project reports. Outputs are defined as distinct achievements directly related (and to some degree traceable) to specific actions coded for in the FIP. Thus this analysis does not attempt to link strategic actions by actors in the FIP to FIP *outcomes* (i.e. measurable change in/on the water). We code for actions, actors and outputs related to either changing policy or practice within the fishery, or associated value chains. Below follow first notes with regard to definitions of variables, assumptions made, and rules for coding of data. Second, we provide tables with all the codes used (for actions/actors/outputs), a description of the code, and examples from the text of how codes were applied.

1. *Actions undertaken by FIPs*

- We code only for (and include in analysis) actions reported in the FIP reports (often referred to as "progress updates", "activities" or "achievements").
- We do not code background information about the fishery or FIP objectives.
- We only include activities listed after the FIP was launched.
- For each recorded occurrence of an action in the FIP reports, we code for action type (see Action code table). Within a FIP there could be multiple actions of any one kind at the same time.

** Note that some variable categories have a built in scale of intensity (e.g. category 6 and 11).

2. Actors:

- For each action we code for the type of actor involved (see Actor code table, A-G).

3. Influencing policy or practice:

Actions and outputs have been divided into those that primarily aim to affect change by targeting either policy or practice. Below we briefly describe the key distinguishing features between these two categories.

Policy related actions (i.e. basic/engaged dialogue-policy) are those actions that aim to achieve a change related to policy and/or regulation, by targeting policy actors/arenas/processes. Examples include engaging in processes to change laws, fishing regulations, quotas, monitoring and actions that aim to improve governance.

Practice related actions (i.e. basic/engaged dialogue-practice) are those actions that aim to achieve a change in practices (not relating to government regulation), by targeting non-governmental actors/arenas/processes (e.g. NGOs, industry). Examples include initiation of traceability schemes – a process which does not always necessitate government enforcement or involvement. Non-government enforced gear changes or changed fishing practices are also included here. Often these practices are used in place of government intervention to affect change in a system. Observer programs that monitor illegal activity will typically be coded as change in practice, unless there is evidence of government regulation or enforcement.

Data dialogues: We have decided to treat dialogues that have data (improvement of, collection of etc.) as their primary focus separate from policy and practice. The reason is that data can, and often is, used for both purposes in the FIPs. However, often the ultimate objective for use of the data discussed in a reported meeting is not clear and to code it as either policy or practice would introduce too much error in the analysis.

4. Level of engagement to affect change:

The actions undertaken to influence either policy or practice are divided into 'basic' or 'engaged' dialogues depending on the assessed level of engagement/effort invested (see full definition below).

Basic dialogue (policy and practice) are either requests, workshops or meetings where there is no documented evidence of deeper discussions, or evidence of sustained dialogue (over time) through e.g. workshop series. Examples include i) reports of discussions where actors are "exploring " new ideas or projects, ii) letters sent by FIPs to government agencies requesting a change in policy, or iii) FIPs attending a meeting organized by someone else and there presenting some ideas/views.

Engaged dialogue *(policy and practice)* are meetings and workshops that include deeper discussions, sustained over time. Examples include evidence of a series of meetings/workshops with clear agendas that suggest two way dialogue and engagement by all parties in the meeting.

5. Outputs:

- We code for output types (See Output code table, 10-12), where outputs are defined as distinct achievements directly related (and to some degree traceable) to specific actions coded for in the FIP (see above).
- We only include outputs that are unlikely (subjectively) to have happened without the involvement of the FIP. This will recognize the "bridging" or "catalyzing" role of FIPs, but does not risk including achievements that cannot, and should not, be attributed to the FIP such as new laws that would have happened regardless.

Policy related outputs (i.e. evidence of changed polices) are outcomes that in some way altered existing policy (e.g. catch monitoring, registration of vessels, by-catch reporting, etc.)

Practice related outputs (i.e. evidence and intensity of changed practice) are those outcomes that in some way altered practices (e.g. including control documents, procurement specs, or similar programs, and observers (both passive and active programs).

Table A. Description and examples of codes for 'Actions'

Number	ACTION TYPE	Code	Theme score examples and details.
1	**Basic dialogue - policy**<br>FIP participants either request policy change (one way communication) or have basic dialogues (workshops or meetings where no additional info is available to assess any deeper discussion around how to affect change) between FIP participants, government organizations, and/or other stakeholders concerning policy.	bdpol	National Fish & Seafood, the first FIP participant, reached out to the Louisiana Department of Wildlife & Fisheries (LDWF) to gain a better understanding of the management of the Louisiana shrimp fishery and to inquire about any existing sustainability assessments that have been completed (Gulf of Mexico Louisiana Shrimp).
2	**Basic dialogue - practice**<br>FIP participants either request a change in practice or have basic dialogues (workshops or	bdpra	Blue Swimmer Crab Public-Private Partnership Kick-Off Meeting", was the launch of a partnership between USAID, ECOFISH, the Province of Bohol, and PACPI to conduct SPR assessment of the blue swimmer crab fishery in Bohol (BSC Philippines).

	meetings where no additional info is available to assess any deeper discussion around how to affect change) between FIP participants and/or other stakeholders concerning practices.		
3	**Dialogue - data**<br>FIP participants either request data improvements or have dialogues between FIP participants and/or other stakeholders concerning data. For example reporting of data results.	ddata	The bilateral workshop was held in Guayaquil, Ecuador in the INP from September 8 to 10, with representatives from the SRP, INP and IMARPE in Peru. Topics discussed included similarities in biological indicators, information exchanges, standardization of research. (Ecuador Mahi Mahi)
4	**Engaged dialogue - policy**<br>Dialogues between FIP participants, government organizations, and/or other stakeholders that show the development of a longer/deeper and sustained discussion or project concerning policy.	engpol	Along the quarter several meetings have been conducted to promote the establishment of a consistent recovery strategy for Grouper fisheries and related species; participating FIP suppliers, fishermen, processors and exporters leaders with local and Federal Authorities leaded by Comite Sistema Producto Mero and accompanied by CeDePesca. Among other initiatives, there is a wide consensus to enhance the closure season from one to two months since 2015 (Mexico grouper).

5	**Engaged dialogue - practice**<br>5.1) Dialogues between FIP participants and/or other stakeholders that show the development of a longer/deeper discussion or project concerning practices. 5.2) pilot projects for practice outputs 5.3) experiential education such as how to use a catch logbook or gear training.	engpra	5.1 At the June Gulf of Mexico Fishery Management Council meeting, Tate of the Reef Fish Shareholders' Alliance announced the exploration of an industry-led Quota Bank, which will primarily address regulatory discard issues in the eastern Gulf of Mexico (US Gulf of Mexico Reef Fish).<br>5.2 experiential education such as how to use a catch logbook or gear training.<br>5.3 The workshops were all well attended and resulted in more than 360 fishermen representing 278 vessels and 94 fishing companies, being trained on the installation, use and maintenance of bycatch reduction technology. This in turn is now helping them to diminish the environmental impacts of their fishing activities (Gulf of California Shrimp)
6.1	**Rally support (ralsup)**<br>Gathering support for the FIP or a project in which FIP has been instrumental by either<br>6.1) requesting funding from specific donor/funder<br>6.2) managing to get approved funding<br>6.3) establishment of permanent funding structure<br><br>This variable has a built in scale of intensity where 6.1 is the lowest and the 6.3 highest.<br>Intensity can be seen as a proxy measure for the likelihood that an outcome will lead to measurable change in outcomes	fndreq	In February, CeDePesca made an informal presentation of a new proposal to the Fishery Management Fund in collaboration with the Fishermen Association of Los Lagos (AG Los Lagos). The proposal focuses on ensuring continuity in this FIP (Chilean Southern Hake).
6.2		fndapp	Brazilian National Congress approved Federal budget, including lobster-monitoring plan requested by the FIP (Brazilian Lobster).
6.3		fndest	Proposals for a shark conservation research project were submitted to funders and 10 cents per pound of all tagged Bycatch of the Day was being donated to fund BCF programs (US Gulf of Mexico Reef Fish).
7.1	**Actions to improve data collection and/or data analysis (datacol)**<br>All kind of data improvements for monitoring and/or management, including data for stock assessment or for example catch logbook. Only used when data is actively collected or analyzed. Categorized into 5 data types:<br>8.1) Stock assessment 8.2) Bycatch 8.3) Compliance 8.4) Catch and effort 8.5) Other (*All data collection is coded as a strategy because*	dstock	A programme of monthly seabob catch sampling (ongoing) undertaken by the industry from 2008 as a key data input to stock Modelling (Guyana seabob).
7.2		dbycat	Bycatch monitoring program was implemented by INAPESCA-Guaymas and local producers in order to identify the species and volumes composition (Sinola Artisanal Shrimp).
7.3		dcomp	While this system was being implemented, the government began to post TED inspection and compliance information on the NOAA Fisheries Southeast Regional Office sea turtle/shrimp fishery website (Gulf of Mexico Louisianan shrimp).

7.4	*we have decided against data collection as an output.)*	dctchef	In October 2012, the update of the catch and effort database continued, incorporating 2012 fishing season information (Panamanian Small Pelagics).
7.5		dother	Used for other types of data collection that does not fit under the other categories or when the data collection is not specified.<br>A short study to evaluate available information on any interactions between the purse seine fishery for tonggol and any small-scale fisheries that take tonggol has been completed. (Thai Tonggol)
8	**Education**<br>Conventional education about for example, management regulations, ecological data or sustainability.	educ	CeDePesca have been developing workshops in order to discuss with local fishermen in different communities: San Felipe, Progreso, Rio Lagartos, Las Coloradas and Chuburná, with the participation of 186 fishermen that were able to hear scientific information of the fishery, a case of successful management in Chile (Mexico Grouper),
9	**Incentives**<br>Marketing incentives from FIP that include, for example, new branding of seafood products to access another market, or other incentives, such as modifying trade barriers or providing subsidies to switch to a different type of gear.	incnt	In March, the brand and logo of the traceability system has been legally registered (Brazilian lobster).

Table B. Description and examples of codes for 'Actors'

Actor category	Code	Explanation	Example
No data/don't know	X	Enough information isn't provided.	
Retailer and 1st tier supplier	A	1st tier suppliers are companies that provide seafood directly to retailers. Retailers are businesses selling seafood products to consumers.	Sea Delight
The rest of the supply chain	B	Actors in the supply chain that are not retailers, 1st tier suppliers, or fishers. For example, processors.	The Indonesia Blue Swimming Crab Producers Association (APRI)
Fishers	C	People involved in the process of catching seafood.	Fishermen; crew, skippers; vessel owners; captains
Government	D	All types of governmental institutions.	Department of Marine Resources (DMR)
Research organization	E	All institutions working with research, like research institutes, universities, and governmental research agencies.	Central Marine Fisheries Research Institute (CMFRI)
NGO	F	Non-Governmental Organizations	WWF
Others	G	Actor types not captured in the categories above.	For example consultants

Table C. Description and examples of codes for 'Outputs', as well as the types of evidence looked for in report to substantiate the claim of an output

Number	Output	Code	Types of evidence	Examples of how to score theme
10.1	**Evidence of changed polices (polout)** Change in fisheries related policies 13.1) fishery management plan 13.2) quotas 13.3) compliance measure and 13.4) limited entry 13.5) others	polout_plan	Fishery management plan made or updated as well as fisheries laws.	The National Tuna Management Plan (NTMP) for Indonesia's tuna fisheries was released by the government of Indonesia in November 2014. (Indonesia Pole and Handline Tuna)
10.2		polout_quot	Quotas systems (Total Allowable Catch).	Establish TAC according to the scientific recommendation. (Chilean Common Hake)
10.3		polout_comply	All types of compliances measures and logbooks.	NOAA Fisheries posted an updated TED inspection and compliance report indicating that fleetwide compliance with federal TED regulations during April 2014–January 2015 ranged from 71 to 100 percent. (Gulf of Mexico Louisiana Shrimp)
10.4		polout_limit	Limited entry to fishery, closed seasons and closed fishing area.	The State Government is strictly enforcing the registration and licensing of fishing vessel to keep a watch on the fleet engaged in the fishery. This would be followed by registration of fishing gears. (India Oil Sardine)
10.5		polout_othr	For example; institutional changes, new management bodies or public consultation.	In September, based on discussions held at the April workshop, the Longline Fisheries Commission was formalized, including representatives of the fisheries authority (ARAP), fishermen, mahi-mahi and tuna exporters, operators of longliners, fisheries researchers, and NGOs.(Panama Pacific Mahi- Mahi and Yellowfin Tuna)

11	**Intensity of changed practices**<br>Level of intensity* of changing practice.<br><br>*intensity can be seen as a proxy measure for the likelihood that an output will lead to measurable change in outcomes	intcgpct	Classify the intensity of changing practices based on the evidence in reports.<br><br>11.1 medium: voluntary changes by fishers or suppliers without compliance by 1st tier supplier.<br>11.2 high: traceability or observer programs with 1st tier supplier oversight	11.1. Hook exchanges were focused on Muisne, with the aim of converting 100% of the vessels in the village…1100 C15 hooks have been installed; fishing gears exchanged from trawl to collapsible trap (2800 traps have been handed over to fishers).(Ecuador Mahi Mahi)<br>11.2. One of programs from 15.1-15.3
12.1	**Evidence of changed practice (praout)**<br>Changes in practices that are likely to have an impact on the sustainability of the fisher. E.g. 15.1)traceability schemes (including control documents, procurement specs, or similar programs); 15.2) observers (both passive and active programs) or 15.3) other examples like gear change	praout_trace	Actions and measurements that increase traceability as<br>12.1.1 control documents (condoc): documents signed by supply chain actors verifying that the product has the sustainability measures defined in the document.<br>12.1.2 procurement specifications (prospec): detailed description characteristics desired in an item to be purchased (e.g. size and weight).<br>12.1.3 other traceability measures (ottrme)	12.1.1 The number of vessels with signed control document increased to 176, representing 25% of the Gulf of California fleet. (Gulf of California Industrial Shrimp)<br>12.1.2 Paul Piazza & Son and Giant Eagle has begun to implement procurement Specifications based on SFP's recommendations. (Texas Shrimp)<br>15.1.3 In March, the brand and logo of the traceability system has been legally registered (Brazilian Lobster).
12.2		praout_ obsv	12.2.1 active observer (actobs) : as people independent of the fishery on board, observing and/or collecting data on by catch, species interactions, etc. or gear inspections.<br>12.2.2 passive observation(pasobs): tools methods such as Vessel Monitoring Systems (VMS) or other surveillance system<br>12.2.3 other examples	12.2.1 In March, Philly Seafood joined the FIP and began to implement gear inspections with their producers. By the end of June, seven of approx.30 vessels in their supply chain had been inspected (Texas shrimp).<br>15.2.2 An electronic monitoring (EM) pilot project was launched by the Ocean Conservancy and the Reef Fish Shareholders' Alliance (RFSA). (Gulf of Mexico Reef Fish)<br>12.2.3

				LTFV begins using radio frequency identification (RFID) tags on frozen tuna, to provide full traceability from the vessel to the end of the supply chain, with plans for full RFID traceability implementation on frozen bigeye and yellowfin tunas by the end of 2015 (Federated Stated of Micronesia Longline Tuna)
12.3		praout_ othr	12.3.1 Other types of changed practices, such as gear change or gear restrictions, bycatch programs, or establishment of fishers' organization	12.3.1 Gulf Wild also implemented a Bycatch of the Day program to market formerly discarded species (Gulf of Mexico Reef Fish).
