# Supplementary material for: Fishery Improvement Projects as a governance tool for fisheries sustainability: A global comparative analysis
Source: PLoS One. 2019 Oct 1;14(10):e0223054. doi: 10.1371/journal.pone.0223054 (PMC6773218; doi:10.1371/journal.pone.0223054)
Supplement: S1 Fig — (PDF) [file pone.0223054.s004.pdf]

S1 Fig. Actions related to data dialogues and data collection reported across regions

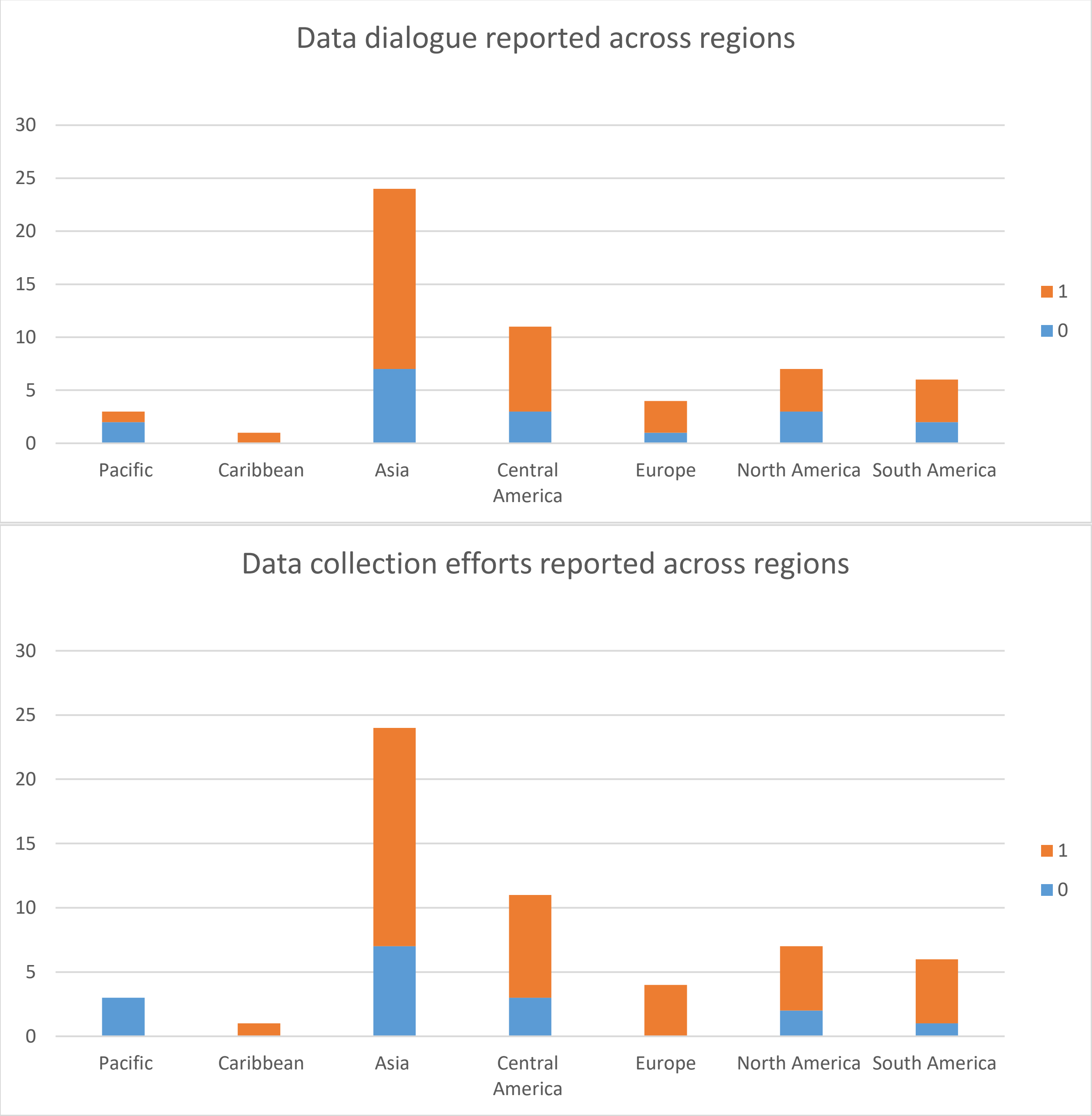

S1 Fig. Top panel shows the number of FIP fisheries employing dialogues around data collection (in orange), and lower panel shows the distribution of actual data collection efforts across FIP fisheries in the same regions (where 1=data collection, 0= no data collection).
