# Supplementary material for: Fishery Improvement Projects as a governance tool for fisheries sustainability: A global comparative analysis
Source: PLoS One. 2019 Oct 1;14(10):e0223054. doi: 10.1371/journal.pone.0223054 (PMC6773218; doi:10.1371/journal.pone.0223054)
Supplement: S1 Table — (PDF) [file pone.0223054.s005.pdf]

S1 Table. Metadata showing database fields available

S1 Table. Metadata showing database fields available. Note that fields with ** were included in the database provided by Sustainable Fisheries Partnership (SFP) in 2015 to the authors. All other fields were added by the authors. The variable FIP status was updated February 2016 by the authors.

Variable name	Description
FIP name**	Name of FIP, per SFP systems
FIP country	Countries associated with the FIP
FIP region	Region associated with the FIP (Africa, Asia, Caribbean, Central America, Europa, Indian Ocean, North America, Oceania, Pacific, South America)
FIP publicly announced (year)**	Year FIP publicly announced
Organization running FIP**	Organization(s) running that FIP
Species (common names)**	Common names of species covered by that FIP
SFP Seafood sector **	The SFP seafood sector is per FishSource database (https://www.fishsource.org/). Note that the sectors Shrimp, Crab and Lobster were made into one category by the authors.
Source	Websites where the FIP report and information were found and downloaded
Date	Date when the FIP reports or information was accessed
FIP Status **	Current status of FIP. If FIP is active (ongoing); inactive (ceased during FIP scoping or terminated); or MSC certified
FIP report category	Reports were classified as 1) assessable [i.e. those that provided sufficient information to allow us to evaluate FIP actions and outputs using our framework] and 2) not assessable [i.e. weak reports or non-reports, those that did not provide sufficient information for analysis] 3) not assessable due to inactivity. Only FIPs in Category 1 'assessable' were analyzed using the codebook (see S1 and S2 Appendices for more info).
FIP species group	Coded FIPs are categorized based on FIP target species. 1 = Crab and Lobster; 2 = Shrimp; 3 = Tuna; 4 = Others

Actor category running FIP	Actor category responsible for organizations running the FIP (Industry, NGO, Fishers, Research organizations, Consultancy)
Codes for actions and actors	Data from coded FIP reports of action and actor-action combinations. 1 means that the actions or actor-action combination occur in the FIP. 0 means that the action or actor-action combination does not occur. NA means that the FIP is not coded. For actor-action combinations codes, the first letters represent the action (e.g. bdpol) and the capital letter in the end represent the actor (e.g. A). For more information about the actions and actor codes see the codebook in S2 Appendix.
bdpol	Basic dialogue - policy
bdpra	Basic dialogue - practice
ddata	Dialogue - data
engpol	Engaged dialogue - policy
engpra	Engaged dialogue - practice
ralsup	Rally support
datacol	Data collection and/or data analysis
edcon	Education
incnt	Incentives
X	No data
A	Retailer and 1st tier supplier
B	The rest of the supply chain
C	Fishers
D	Government
E	Research organization
F	NGO
G	Others
Codes for outputs	Data from coded FIP reports of outputs. 1 means that the output occurs in the FIP. 0 means that the output does not occur. NA means that the FIP is not coded. For more information about the outputs codes see the codebook in S2 Appendix.
polout	Evidence of changed polices
praout	Evidence of changed practice

polout_cpplan	Fishery management plan
polout_cpquot	Quotas systems
polout_cpcomply	Compliances measures
polout_cplimit	Limited entry to fishery
polout_others	Other changes in fisheries related policies
praout_cprtrace	Traceability schemes (including control documents, procurement specs, or similar programs)
praout_ obsv	Observers (both passive and active programs)
praout_ othr	Other examples like gear change
