# Supplementary material for: Fishery Improvement Projects as a governance tool for fisheries sustainability: A global comparative analysis
Source: PLoS One. 2019 Oct 1;14(10):e0223054. doi: 10.1371/journal.pone.0223054 (PMC6773218; doi:10.1371/journal.pone.0223054)
Supplement: S2 Table — (PDF) [file pone.0223054.s006.pdf]

S2 Table. Cross-tabulation of FIP actor type by action

S2 Table. **Cross-tabulation of FIP actor type by action.** Actions are listed with each fishery in the leftmost column, and actor types are listed as headers of subsequent columns. Each actor-action combination is shown as a percentage of all coded records of actor-action combinations for that particular fishery. Each fishery type has been conditionally formatted to highlight (through color intensity) which actor-action combination is most prevalent in this fishery context. 'Others' represents a mix of other actor types, but consultants are overrepresented in this category. 'No data' represents reported actions where no specific actors were identified as contributing.

**All fisheries**	**Retail/1st tier**	**Rest of sup chain**	**Fishers**	**Government**	**Research Org**	**NGO**	**Other**	**No data**
Basic dialouge policy	8	20	13	24	8	18	7	2
Basic dialouge practice	10	23	18	10	4	21	11	4
Data dialouge	6	16	12	17	16	20	11	2
Engaged dialouge policy	10	22	8	29	6	22	2	0
Engaged dialouge pratice	8	20	26	14	6	16	9	1
Rally support	11	22	6	6	6	17	33	0
Data collection	9	25	15	12	18	11	9	1
Education	9	18	33	9	3	21	6	0
**Crab/lobster**								
Basic dialouge policy	11	19	0	22	15	19	11	4
Basic dialouge practice	15	20	10	15	5	20	15	0
Data dialouge	0	12	8	23	19	19	19	0
Engaged dialouge policy	13	31	0	25	6	19	6	0
Engaged dialouge pratice	0	18	18	9	9	27	18	0
Rally support	0	25	0	13	0	13	50	0
Data collection	4	24	4	16	28	4	20	0
Education	7	27	20	13	7	20	7	0
**Shrimp**								
Basic dialouge policy	11	21	16	32	0	16	5	0
Basic dialouge practice	20	20	0	0	0	40	20	0
Data dialouge	17	22	11	11	17	11	11	0
Engaged dialouge policy	0	20	20	20	20	20	0	0
Engaged dialouge pratice	14	14	24	14	10	10	14	0
Rally support	100	0	0	0	0	0	0	0
Data collection	19	25	13	13	13	6	13	0
Education	17	0	50	0	0	17	17	0
**Tuna**								
Basic dialouge policy	5	26	18	21	8	13	8	3
Basic dialouge practice	5	37	21	5	0	11	16	5
Data dialouge	3	16	10	23	13	16	16	3
Engaged dialouge policy	17	25	8	33	0	17	0	0
Engaged dialouge pratice	10	23	29	16	6	6	6	3
Rally support	0	33	0	0	0	33	33	0
Data collection	0	35	18	12	12	12	6	6
Education	0	20	60	0	0	20	0	0
**Others**								
Basic dialouge policy	8	16	16	26	8	24	3	0
Basic dialouge practice	7	17	24	10	7	24	3	7
Data dialouge	8	16	16	14	16	25	4	2
Engaged dialouge policy	6	13	13	31	6	31	0	0
Engaged dialouge pratice	4	22	26	13	0	30	4	0
Rally support	17	17	17	0	17	17	17	0
Data collection	13	19	23	10	16	19	0	0
Education	14	14	29	14	0	29	0	0
